# An atypical metastatic pathway: Bilateral inguinal lymphadenopathy as the initial presentation of papillary thyroid carcinoma

**DOI:** 10.1016/j.ijscr.2025.111189

**Published:** 2025-03-26

**Authors:** Alsadig Suliman, Rawan Mohamedosman, Mohamed Soud, Hiba Suliman, Alsadig Suliman

**Affiliations:** a Sudan Medical Specialization Board, Department of General Surgery, Khartoum, Khartoum State, Sudan; b University of Khartoum, Faculty of Medicine, General Surgery Department, Khartoum, Khartoum State, Sudan; c University of Gezira, Faculty of Medicine, Wad Madani, Gezira State, Sudan; d Wad Medani College of Medical Sciences & Technology, Wad Madani, Gezira State, Sudan

**Keywords:** Case report, Papillary thyroid carcinoma, Inguinal lymphadenopathy, Psammoma bodies, Thyroid nodule, Sudan

## Abstract

**Introduction and importance:**

Papillary thyroid carcinoma (PTC) is known to primarily manifest as a thyroid nodule with regional lymph node metastasis. Distant metastasis in PTC is rare, making its diagnosis challenging when presenting atypically.

**Case presentation:**

A 47-year-old Sudanese female was evaluated for bilateral inguinal lymphadenopathy and significant weight loss. Extensive diagnostic efforts, including abdominal imaging and thyroid function tests, showed no abnormalities and no palpable thyroid nodules. However, a biopsy of the inguinal lymph nodes (ILNs) revealed psammoma bodies, confirming PTC. Further evaluation detected a 1 cm thyroid nodule.

**Clinical discussion:**

This case highlights the unusual presentation of PTC with distant metastasis to the ILNs without the typical presence of a thyroid nodule or cervical lymphadenopathy. The discovery underscores the importance of considering PTC in differential diagnoses for unexplained inguinal lymphadenopathy, emphasizing the need for thorough evaluation in atypical presentations.

**Conclusion:**

The rarity of inguinal lymphadenopathy as an initial presentation of PTC demonstrates significant diagnostic challenges. This case emphasizes the necessity of including PTC in the differential diagnosis for inguinal lymphadenopathy, advocating for comprehensive diagnostic strategies to manage and improve outcomes in such atypical cases. The patient's refusal of surgery and subsequent loss to follow-up culminating in undocumented death further complicate management in such rare presentations.

## Highlights

• A 47-year-old female presented with bilateral inguinal lymphadenopathy and significant weight loss.
• Despite normal thyroid function and no palpable nodules, biopsy revealed psammoma bodies confirming PTC.
• Metastasis of papillary thyroid carcinoma to inguinal lymph nodes is extremely rare.
• PTC should be considered in cases of unexplained inguinal lymphadenopathy without typical thyroid signs.
• The patient declined surgery and was lost to follow-up, highlighting management challenges in rare cases.

## 1 Introduction

Papillary thyroid carcinoma (PTC) is the second most common thyroid cancer reported among the Sudanese population, affecting females more than males, with a mean age of 53.4 (SD 13.7) [1]. PTC typically presents as a thyroid nodule initially, with regional lymph node metastasis occurring less frequently in about 30–80 % of patients [2,3]. However, non-regional lymph node involvement, especially below the diaphragm, as seen in our case, is exceedingly rare [4]. Distant metastasis occurs in approximately 5 % of patients, with the lungs, bones, and brain being the most frequent sites. Metastasis to the inguinal lymph nodes (ILNs) is exceptionally rare and often not considered in the initial differential diagnosis [5,6]. The gold standard for diagnosing PTC is through ultrasound and fine needle aspiration. Psammoma bodies, which are microcalcifications found in histopathology, are the most characteristic finding of malignancy, with a positive predictive value of up to 95 % [7]. In this study, the patient presented with bilateral inguinal lymphadenopathy. Following bilateral lymph node biopsy, histopathology showed PTC despite having no clinical or radiological findings suggestive of thyroid cancer. Since this appears to be a rare presentation in the literature, we reported this case to add to the existing information and emphasize the importance of considering PTC in the differential diagnosis of inguinal lymphadenopathy [8]. This work has been reported in line with the SCARE criteria [9].

## 2 Case presentation

A 47-year-old Sudanese female farmer, with no prior history of chronic illnesses, malignancies, recent infections, or thyroid disorders, presented with bilateral inguinal lymphadenopathy of two months' duration. She reported significant weight loss during this period but denied fever, night sweats, chills, dyspnea, cough, arthralgia, rashes, or other systemic symptoms. She had no history of prior surgeries, medication use, smoking, alcohol consumption, radiation exposure, or sexually transmitted infections (STIs). Additionally, there was no family history of thyroid cancer or other malignancies.

On physical examination, the patient had multiple firm, non-tender, mobile ILNs of varying sizes bilaterally, with no overlying skin changes. The rest of the systemic examination, including the thyroid and cervical regions, was unremarkable, with no palpable thyroid nodules or cervical lymphadenopathy. There was no hepatosplenomegaly or uveitis.

Initial routine laboratory tests, inflammatory markers, and infectious disease screenings were unremarkable (Table 1). Given the persistent and unexplained nature of the lymphadenopathy, further evaluation was warranted. A contrast-enhanced CT scan of the abdomen and pelvis showed no evidence of primary malignancies, with the ovaries, pelvic organs, and other abdominal organs appearing normal.

**Table 1:** Laboratory investigations.

Investigation	Findings	Reference range
Complete blood count (CBC)		
Hemoglobin (Hb)	13.2 g/dL	12.0–15.5 g/dL
White blood cell count (WBC)	6.8 × 10^9^/L	4.0–11.0 × 10^9^/L
Platelet count	260 × 10^9^/L	150–400 × 10^9^/L
Peripheral blood smear	Normal	–
Inflammatory markers		
C-reactive protein (CRP)	1.5 mg/L	<5 mg/L
Erythrocyte sedimentation rate (ESR)	10 mm/h	<20 mm/h
Infectious disease screening		
HIV screening	Negative	–
Syphilis serology (RPR/VDRL)	Negative	–
Hepatitis B surface antigen (HBsAg)	Negative	–
Hepatitis C antibody (Anti-HCV)	Negative	–
Thyroid tests		
Thyroid-stimulating hormone (TSH)	1.8 mIU/L	0.5–5.0 mIU/L
Free T3 (FT3)	3.2 pg/mL	2.3–4.2 pg/mL
Free T4 (FT4)	1.3 ng/dL	0.8–1.8 ng/dL
Liver function tests (LFTs)		
Alanine aminotransferase (ALT)	22 U/L	7–56 U/L
Aspartate aminotransferase (AST)	18 U/L	10–40 U/L
Alkaline phosphatase (ALP)	78 U/L	30–120 U/L
Total bilirubin	0.8 mg/dL	0.2–1.2 mg/dL
Albumin	4.2 g/dL	3.5–5.0 g/dL
Renal function tests (RFTs)		
Blood urea nitrogen (BUN)	14 mg/dL	7–20 mg/dL
Serum creatinine	0.9 mg/dL	0.6–1.3 mg/dL
Serum calcium	9.2 mg/dL	8.5–10.5 mg/dL
Parathyroid hormone (PTH)	38 pg/mL	10–65 pg/mL

**Fig. 1:**
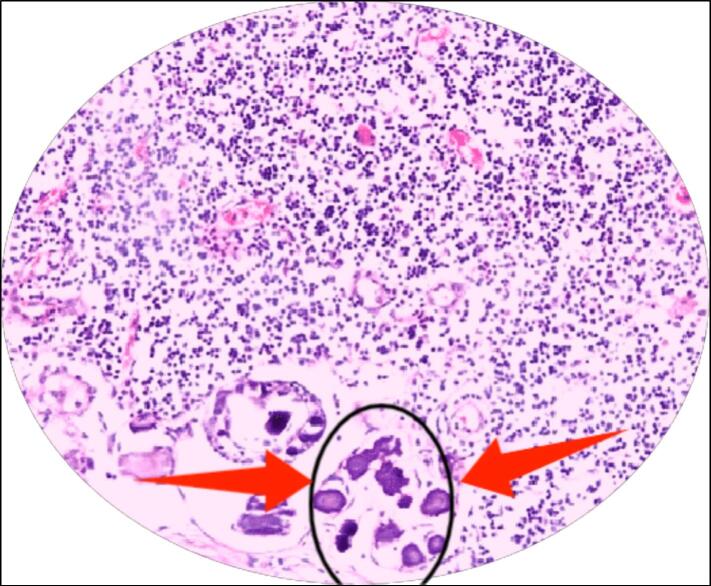
Histopathology of the inguinal lymph node showing psammoma bodies stained with H&E at 40× magnification.

Due to the ongoing persistence of lymphadenopathy and the absence of clear clinical findings, an incisional biopsy of the right ILN was performed. Histopathological examination revealed psammoma bodies and follicular structures consistent with metastatic PTC (Fig. 1). These unexpected findings prompted further investigation.

**Fig. 2:**
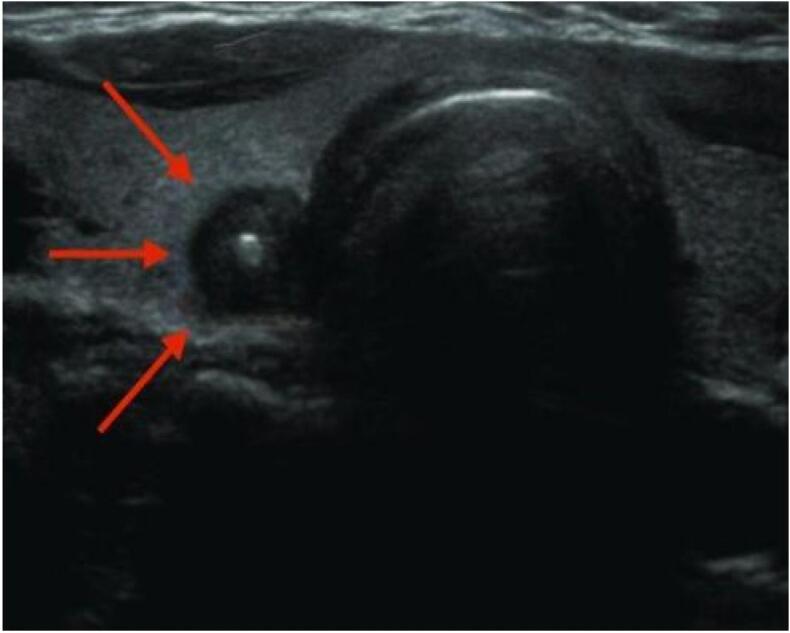
Neck ultrasound showing a 1 cm thyroid nodule (indicated by arrows) with no other abnormalities detected.

Following the identification of psammoma bodies in the right ILN biopsy, a neck ultrasound was performed, revealing a 1 cm hypoechoic thyroid nodule with irregular margins and features suggestive of PTC (Fig. 2). Given the histopathological confirmation of PTC in the inguinal lymph node, fine needle aspiration (FNA) of the thyroid nodule was not pursued, as ultrasound-guided thyroid biopsy was not available in our setting.

To strengthen diagnostic confidence, a clinical meeting was held to discuss the atypical presentation of metastatic PTC with inguinal rather than cervical lymph node involvement. For diagnostic cross-validation, a repeat biopsy of the left ILN was performed to confirm histopathological findings in a separate lymphatic basin. The specimens were sent for independent review, which confirmed psammoma bodies consistent with metastatic PTC. Identical histopathological findings in both lymph nodes, along with concordance from a separate laboratory, reinforced the final diagnosis and ruled out alternative etiologies. A multidisciplinary team consulted and recommended a total thyroidectomy after carefully counseling the patient about the findings and potential implications. However, the patient expressed concerns about surgery, citing possible complications and a preference for alternative treatments due to cultural beliefs. After thorough discussions regarding the risks and benefits, the patient opted for an informed refusal of surgery. Her decision and the reasons for it were extensively documented.

Following the patient's decision to decline surgery, our clinical team made significant efforts to schedule multiple follow-up appointments to monitor her condition and provide ongoing support. However, maintaining consistent communication proved challenging due to the inherent limitations of our low-resource setting. The patient's limited and unreliable contact information further complicated these efforts, impeding our ability to ensure continuous care.

Regrettably, after two months of unsuccessful attempts to reconnect with her, we were informed by community intermediaries that the patient had passed away. The details surrounding her death remain unclear, as the cause of death was not documented, and no further information was forthcoming.

## 3 Discussion

Although PTC is generally considered a low-grade tumor with a favorable prognosis when confined to the thyroid gland or cervical lymph nodes, distant metastasis significantly worsens outcomes, with mortality rates ranging from 24 % to 76 %. Specifically, distant lymph node metastasis is associated with a mortality rate of 26.7 % [[10], [11], [12]]. Occult metastatic PTC has been reported frequently in Asia, with few cases documented in Africa. Metastasis of PTC to the ILNs is exceptionally rare, with our literature search revealing only two previously reported cases.

The differential diagnosis for bilateral inguinal lymphadenopathy is broad and includes infectious, hematologic, and metastatic neoplastic processes, necessitating a systematic approach to exclusion [[13], [14], [15], [16]]. Tuberculosis remains a prevalent cause of persistent lymphadenopathy in endemic regions; however, the absence of caseating granulomas on histopathology, negative inflammatory markers, and unremarkable imaging findings ruled out tuberculous lymphadenitis [13]. Similarly, STIs such as syphilis and HIV frequently present with inguinal lymphadenopathy, but negative serological tests and the lack of systemic symptoms excluded these infectious causes [14]. Hematologic malignancies, particularly lymphoma and leukemia, were strongly considered given the persistent lymphadenopathy [15]. However, normal levels of lactate dehydrogenase (LDH), along with an unremarkable peripheral blood smear, significantly reduced the likelihood of a primary lymphoproliferative disorder. Additionally, comprehensive abdominal and pelvic imaging ruled out pelvic or lower abdominal malignancies, which commonly metastasize to the ILNs [19]. Genetic testing aids in diagnosing PTC by detecting mutations like BRAF V600E and RET/PTC rearrangements, which influence prognosis and treatment. BRAF V600E is present in 43–88 % of PTC cases. However, due to resource limitations, we were unable to perform it in this case [17].

Struma ovarii, a specialized ovarian teratoma composed of thyroid tissue, was considered as a rare differential diagnosis. While malignant struma ovarii can metastasize via lymphatic and hematogenous routes, it typically remains confined to pelvic and para-aortic lymph nodes, with no documented cases of metastasis to the ILNs [18,19]. Furthermore, a CT scan of the abdomen and pelvis revealed normal ovarian structures, making this diagnosis unlikely. The presence of psammoma bodies in the ILNs biopsy was a critical diagnostic clue, as these structures are highly specific for PTC.

Previously published cases of ILN metastasis from thyroid cancer remain exceedingly rare. A case reported in Russia in 1986 described an isolated lesion in the ILN as the first sign of PTC [20]. However, this case lacked details on disease progression and patient outcomes. Mari et al. reported a male patient diagnosed with PTC who developed ipsilateral ILN metastasis, but unlike our case, he had an underlying multinodular goiter and concurrent lung metastases [8]. Similarly, an Italian case described a 63-year-old man with metastatic medullary thyroid carcinoma (MTC) involving the cervical lymph nodes, lungs, vertebrae, sacrum, and testis, with histological confirmation of MTC in both the right ILN and testicular nodule [21]. These cases differ from ours in that their metastatic patterns included other organ systems, whereas our patient initially presented with bilateral ILN metastases without prior thyroid disease or detectable distant metastases at diagnosis.

Additionally, a case from India described a woman diagnosed with follicular thyroid cancer who developed ipsilateral ILN metastasis after total thyroidectomy. However, unlike our patient, she exhibited widespread metastatic disease involving the iliac pelvic bone, gluteal muscles, scalp, spine, chest wall, and mediastinal lymph nodes [4]. Given the unusual ILN involvement in thyroid cancer, Damle et al. proposed struma ovarii as a differential diagnosis in a similar case [4]. However, as in our case, imaging findings did not support this diagnosis, reinforcing the final confirmation of metastatic PTC.

Our case is unique due to the bilateral ILN involvement as the sole initial manifestation of PTC, with no prior thyroid disease or detectable metastatic spread at the time of diagnosis. The patient's decision to decline surgery due to personal and cultural beliefs, combined with the challenges of follow-up in a low-resource setting, underscores the complexities of managing such rare presentations.

## 4 Conclusion

Inguinal lymphadenopathy as the sole presentation of PTC is exceptionally rare and presents significant diagnostic challenges. This case underscores the importance of considering PTC in the differential diagnosis for unexplained inguinal lymphadenopathy, even when typical thyroid symptoms and nodules are absent. Early recognition and appropriate management are crucial for improving patient outcomes in such atypical presentations. Further research is needed to explore the mechanisms behind these unusual metastatic patterns and to enhance diagnostic strategies.

## Author contribution

• Alsadig Suliman (Corresponding Author)
  o Conceptualization and study design
  o Data interpretation
  o Writing original draft and revising the manuscript
  o Final approval of the version to be published.
  o Review and editing of the manuscript.
  o Conducting literature review
• Rawan Mohamedosman
  o Data collection
  o Conducting literature review
  o Writing sections of the manuscript
  o Review and editing of the manuscript.
• Mohamed Soud
  o Data collection
  o Conducting literature review
  o Drafting parts of the manuscript
• Hiba Suliman
  o Data collection
  o Picture collection
  o Review and editing of the manuscript.

## Consent

Written informed consent was obtained from the patient's family for publication of this case report and accompanying images. A copy of the written consent is available for review by the Editor-in-Chief of this journal on request.

## Ethical approval

This case report did not require ethics approval as it involves a single patient case that does not constitute research according to our institution's guidelines. The Author Form Institutional Review Board (IRB) of Sudan Medical specialization board has confirmed that ethical approval is not necessary for case reports of this nature. Informed consent for publication, including the use of images, was obtained from the patient.

## Guarantor

Alsadig Suliman accepts full responsibility for the work and the conduct of the study, had access to the data, and controlled the decision to publish.

## Research registration number

Not applicable – this submission is a case report and not a registered study.

## Funding

No extramural funds were used to support this case report.

## Conflict of interest statement

The authors declare no conflicts of interest. The authors confirm the accuracy of the data.
